# Modular structures and the delivery of inpatient care in hospitals: a Network Science perspective on healthcare function and dysfunction

**DOI:** 10.1186/s12913-022-08865-8

**Published:** 2022-12-10

**Authors:** David I. Ben-Tovim, Mariusz Bajger, Viet Duong Bui, Shaowen Qin, Campbell H. Thompson

**Affiliations:** 1 College of Medicine and Public Health, Flinders University, 5042 Bedford Park, SA Australia; 2 College of Science and Engineering, Flinders University, 5042 Tonsley, SA Australia; 3 Royal Adelaide Hospital, 5000 Adelaide, SA Australia

**Keywords:** Network graphs, Network science, Modularity, Healthcare services provision, Congestion, Covid-19

## Abstract

**Background:**

Reinforced by the COVID-19 pandemic, the capacity of health systems to cope with increasing healthcare demands has been an abiding concern of both governments and the public. Health systems are made up from non-identical human and physical components interacting in diverse ways in varying locations. It is challenging to represent the function and dysfunction of such systems in a scientific manner. We describe a Network Science approach to that dilemma.

General hospitals with large emergency caseloads are the resource intensive components of health systems. We propose that the care-delivery services in such entities are modular, and that their structure and function can be usefully analysed by contemporary Network Science. We explore that possibility in a study of Australian hospitals during 2019 and 2020.

**Methods:**

We accessed monthly snapshots of whole of hospital administrative patient level data in two general hospitals during 2019 and 2020. We represented the organisations inpatient services as network graphs and explored their graph structural characteristics using the Louvain algorithm and other methods. We related graph topological features to aspects of observable function and dysfunction in the delivery of care.

**Results:**

We constructed a series of whole of institution bipartite hospital graphs with clinical unit and labelled wards as nodes, and patients treated by units in particular wards as edges. Examples of the graphs are provided. Algorithmic identification of community structures confirmed the modular structure of the graphs. Their functional implications were readily identified by domain experts. Topological graph features could be related to functional and dysfunctional issues such as COVID-19 related service changes and levels of hospital congestion.

**Discussion and conclusions:**

Contemporary Network Science is one of the fastest growing areas of current scientific and technical advance. Network Science confirms the modular nature of healthcare service structures. It holds considerable promise for understanding function and dysfunction in healthcare systems, and for reconceptualising issues such as hospital capacity in new and interesting ways.

## Background

An abiding concern during the COVID-19 pandemic has been the capacity of health systems to cope with the demands of people infected with SARS-CoV-2. There has been anxiety that hospitals, overwhelmed by demands for care, might break under the strain. However, hospitals are not brittle objects. Nor are they static fortified positions that can be overwhelmed by an encroaching force. They are complicated, evolved, human artefacts whose designs include a range of mechanisms [1] to balance institutional viability and the, at times, competing demands of individual patients whose needs are further championed by the staff caring for them.

Biomedical research and practice is appropriately concerned with identifying the right biomedical and psychosocial care for patients and communities. However, right care is the product of a system whose elements include providing right care, to the right patient, at the right time, in the right place, and, whenever possible, right first time. In that context, the provision of care is as much a function of the interactions between the components that make up a system of care as it is of the technical competencies required. But the complexity of the demands, and the diversity of the necessary responses, mean that it is challenging to represent the interactions involved in a scientifically robust manner [2] and to develop metrics of system level functioning that span periods when the system is running smoothly and when it is dysfunctional. For example, problems with an Emergency Department were leading to failures to meet specific targets for initiation of meaningful treatment [3]. There were measurable delays in admitting patients to the body of the hospital, and there were measurable increases in the number of ambulance queuing to offload patients. None of those outcome indicators provided any insight into the organisation of the resources for patients within a Department when it was running smoothly or under strain. When internal organisational problems that had emerged during a period of strain were resolved, the functioning of the Department immediately improved [1, 3] without any new resources being required. A five year review demonstrated that the improvements had been maintained [1]. It is organisation within systems of care, and the potential impact of those organised systems on patient related outcomes, that this paper is concerned with.

The aims of our program of work have therefore been, first, to create a quantitative, structured, representation of how the human and physical resources involved in delivering patient care work together [4] as a functioning whole. Then, to explore the relationship between that representation and other measures of hospital function and dysfunction. Due to the complexity of the systems involved, a degree of abstraction is a required. Nevertheless, we have tried to remain mindful of our cumulative experience redesigning real-world healthcare systems [3, 5, 6].

In the current study, whilst bearing the aims in mind, we have chosen to focus on a component part of health service provision, rather than a total service. A Public hospital is not a single functional entity. For example, out-patient clinic-type services work differently to inpatient services. Same-day procedural activities are different again. The primary focus of our current analysis has been overnight stay services, an important source of public concern when there is evidence of dysfunction. We conceptualise overnight care as the product of interactions within and between three general functional layers.

A tripartite structure is common in analyses of complicated systems with fixed elements subject to variable demands. Examples include traditional telephone, and electrical transmission, systems [7]. Tripartite structures have also been widely adopted in the allied field of healthcare operational research [8]. In the tripartite structure proposed here, the primary, or base, layer (corresponding to the operational layer in operational research) is made up from direct patient-care-provider contacts. Then there is a top layer of political, bureaucratic, and managerial, policy and control over human and physical resources (corresponding to the strategic level in operational research), incorporating managerial constructs such as allocating services to Divisions or Departments, as seen in organisation charts. Finally, corresponding to the tactical layer in operations research, we postulate a functional middle layer made up from the day-to-day organisation and logistics of the human and physical interactions involved in supporting the direct delivery of care. It is within this functional middle layer that pressures on institutional overnight stay capacity are likely to be both transmitted (e.g. COVID-19 is causing an increased demand for personal protective equipment and ventilators) and buffered (e.g. how can we best re-organise our resources to meet surges in demand?). As such, this middle layer is our layer of interest, and the focus of our efforts at quantifiable, and generalisable, representations.

The current study is based on the proposition that the organisation of healthcare functional structures can be best understood using the construct of modularity, and analysed using the scientific and technical approaches of contemporary Network Science. Using a hypothesis testing format, the null hypothesis is that network graphs of inpatient healthcare processes have a random graph structure. The test hypothesis is that the network graphs [7] have a structure that is unlikely to have occurred by chance.

### Modularity and Network Science

Some years ago, Simon [9] expounded a general theory for the organisation of complicated biological and human systems. He argued that many complicated systems were made up from subsystems which in turn contain their own subsystems, in a hierarchical fashion. His key assertion was that interactions within such systems would be made up from semi-autonomous interacting near-decomposable, or modular, entities. Important advantages of modular systems are that the impact of breakdowns can be contained within modular components and that modular systems can adapt to changing circumstances a module at a time, without disrupting overall system integrity. Our preceding experience redesigning healthcare processes emphasised the possibility that Simon’s work would be relevant to healthcare [1]. Baldwin and Clark [10] have provided a quasi-operational definition of a module based on Simon’s concept of near-decomposability. “A module is a unit whose structural elements are powerfully connected among themselves and relatively weakly connected to elements in other units. Clearly there are degrees of connection, thus there are gradations of modularity” ([10] p.63). Hierarchical modularity has now been identified as an organising principle in a wide range of biological and human created systems [10]. Whilst modularity has been explored in healthcare, this has been mainly in regards to ‘plug and play’ modular component interoperability and narrow focussed improvements [11]. By contrast, an example of the focus of the current program of work can be seen by analogy with changes in the area of interventional cardiology.

The evolution of cardiac stents from bare metal to drug-eluting has been an important therapeutic advance. The new stents could be incorporated into everyday use as a specific change in a restricted area of practice, that is, as an intramodular process change. The take-up at any scale of another important cardiac technology, implantable defibrillators, involves a much larger system redesign incorporating a number of modular components [12, 13] and their interactions. It is the development of quantitative representations of inter-modular functional structures and their interactions, that is of particular interest here. However, whilst modularity can be described in both words, and as symbolic matrices of various kinds [14, 15] it had been hard to quantify until advances in Network Science have enabled modularity to be studied as a quantitative system metric [16].

Network Science has been described as relational science [17], a science of how the components of systems work together [18]. As a scientific discipline, Network Science rests on certain basic aspects of the properties of network graphs, and it is important to be aware of these basic aspects when considering the products of Network Science analyses. Networks represent phenomena of interest as a series of points, called nodes or vertices, linked in pairs by edges, lines that represent whatever links the nodes. In the production of a network graph [17], the data is formatted, then analysed, as an adjacency matrix in which nodes form both row and column headers. The matrix cells identify whether a pair of nodes do, or do not, interact. When interaction patterns within adjacency matrices are mapped as a graph on a plane, the resultant figure has topological properties. That is, its spatial and geometric relationships are preserved under continuous deformations such as stretching, twisting, or bending, but not tearing apart. A range of mathematical tools can be employed to represent, then analyse, the interconnection between the entities that make up the topologically described networks [7, 19].

An important feature of network graphs of complicated systems is that the structure of the graphs can be tested against the null hypothesis that the graphs depicts a random [20], or null-state system. As described by Erdős and Rény [20], in random graphs the nodes connect at random. As a result, the likelihood of having an edge between any pair of nodes simply follows the laws of probabilities and the pattern of interconnections follow a Gaussian, or normal distribution. The probability (or rather, the improbability) of the chance emergence of members of families of graphs with various kinds of specific non-normal features can then be calculated.

Newman [18] has made it clear that modularity as described by Simon [9] has a counterpart in topological modularity. It has been found that many topologies of sparse systems (systems where the number of edges is of the same order of magnitude as the number of nodes) have an internal structure in which clusters, or communities, of nodes can be identified [16]. As premised by Simon [9] the nodes within communities are densely interconnected with each other, and sparsely interconnected with nodes in other communities. Newman developed a quantitative parameter that specifies the overall modularity of a network graph. Modularity values approaching 1, the maximum, imply a network with a very strong modular structure. In practice, values of 0.3 to 0.7 are common in networks with recognisable communities [18]. A variety of algorithms for community detection are available [16]; modularity as a graded phenomenon has been demonstrated in network graphs of a wide variety of systems [16].

The depiction of healthcare systems as network graphs is not new. A number of healthcare related studies based on network graphs have been published. This includes a substantial literature of studies using social network analyses [21–23]. Recently, healthcare-related studies [24, 25]based on the more quantitative contributions of statistical physicists to Network Science have begun to emerge. A number of these have been predominantly concerned with movements of patients across networks [26–28]. As yet, there has been limited work using community detection methods to describe the functional organisation of healthcare delivery at the operational level. This deficiency [24] is addressed in the studies described below.

## Methods

### Constructing healthcare network graphs

Networks may be represented by many different types of graphs. We constructed a series of undirected bipartite graphs [29, 30] (graphs made up from two types of nodes) using named wards and clinical units as nodes. The patients linking the units and wards that were related to their care formed the edges.

A designated ward not only identifies a set of physical resources; it represents the nursing and other ward specific groups of staff involved in the delivery of the care of the patients managed in that ward. The doctors and allied health practitioners who take responsibility for specific areas of diagnosis, care planning, and therapy, commonly work in named clinical units. Those units may provide care in a variety of locations. A clinical unit might function in one ward only (e.g. a single geriatric team might provide inpatient care in a Geriatric Evaluation and Management designated Ward) or in multiple wards (a cardiology unit might care for patients in a Coronary Care Unit and two cardiac-designated wards).Contrawise, a designated ward, and its geographically based staff, may host patients under the care of one, or many, clinical units.

Network graphs may be undirected, or directed if there is a dependency that subordinates one node to another. However, both types of graphs can be analysed in similar ways. In hospitals and health services, patient care is the product of teamwork between many different groups of staffs: that teamwork is best represented by undirected interactions.

All the analyses reported here were undertaken on anonymised data sets. The studies were performed in conformity to an institutional ethics review by the relevant health authority, as described below.

Network graphs are arithmetic products. Their utility depends on their interpretability by domain experts. A series of meetings were convened with a multi-disciplinary range of healthcare domain experts who reviewed the bipartite undirected graphs. Their comments led to a novel analysis of the graphs’ modular structures.

### Data sources

The current studies [25] made use of anonymised hospital data derived from mandated patient level data systems (patient administrative data sets). In Australia, those data systems follow detailed national guidelines [31]. The anonymisation process prevents the identification of individual patients, but data sets of this kind contain patient related data. For privacy reasons, they are not readily made available for public use.

Anonymised administrative data sets for two Australian public general hospitals, Hospital 1 and 2, were accessed. Both hospitals provided a range of secondary and tertiary care services to substantial catchment populations. Hospital 2 also provides a range of state-wide super-speciality services. It does not provide paediatric, gynaecological or obstetrics services. Both hospitals collect mandated inpatient administrative data as described above. Hospital 1 had also developed a searchable patient journey data base that overlays inpatient data with a computerised time and location stamp each time a patient is moved between locations within the hospital. This allows a ward and clinical unit to be identified at the point that data is extracted. The treating wards and units in Hospital 2 were as recorded at the point of discharge. For both hospitals, the data covered the whole of 2019, and up to September in 2020. The data collection could not be extended in Hospital 1. After that time any data extraction procedures would have had to be undertaken by analysts who were required to work exclusively on immediate COVID pandemic issues. For Hospital 2, the data collection was extended to cover the remaining months of 2020.

### Adjacency matrices, network graphs and community detection :. identifying functional structures and network graph characteristics

For Hospital 1 and 2, adjacency matrices were developed based on each monthly snapshot. The snapshots were also merged for calendar year matrices. In the adjacency matrices, the complete set of wards and units formed both the column and the row headers, and the presence or absence of shared patients formed the cell content. This was not an accumulative procedure; only the link at the moment of overall data extraction was recorded. The data extraction was based on the same date each month, randomising the data in relation to the day of the week. Whilst data was available to weight the edges in relation to the numbers of patients involved, unless otherwise indicated, as previously stated, the studies were conducted on unweighted, undirected data.

The matrix data was analysed using specialised open-source software Gephi [32] (Version 0.9.2). The widely used Louvain community detection algorithm [33] for detecting modules was applied, and the resultant graphs displayed using the ForceAtlas2 algorithm [34]. ForceAtlas2 is a force directed layout program within Gephi, used to spatialize a network. The Gephi program allows for the computation of a variety of graph parameters, including the modularity parameter.

Network graphs of the monthly snapshots and of the composite calendar year data bases were prepared for each Hospital. Graphs were first produced for the whole population of nodes and edges. In due course, subtraction graphs were produced in which emergency admission only, or elective admission only, graphs were created.

### Relating graph identified functional structures to a measure of hospital dysfunction

The domain experts reviewed the graphs for the whole population of nodes. They noted that the graphs, which were readily interpretable, pointed to a heterogenous nature for the modular structures within the networks. A minority of modules appeared to demonstrate a greater degree of internal coherence than the remaining modules. The possibility was also raised that variations in overall graph coherence might relate to periods of overcrowding in the Emergency Department. These issues were further explored in Hospital 1, whose data included a surrogate measure of the outcome of hospital congestion episodes.

A network graph is a memoryless figure. To develop a longitudinal representation of variations in network make-up, links between graphs were required that would provide some insight into the behaviour, over time, of the differing modules. Pairs of recurring ward unit combinations were used to link a range of modules across graphs, making it possible to identify the temporal variability of the range of modular structures in each hospital (full details of the procedure involved are available from the corresponding author on request).

The Coefficient of Variation (cv) for identified modular structures in each hospital was computed, and presented in Table 1. The cv is the ratio of the standard deviation of a set of values in a data set to their mean value. Empirically, we took a cv value of 0.2 as a threshold separating what we then termed high coherence modules (cv < 0.2), from the remaining modules. There were three such high coherence modules in each hospital.

The data base for the snapshots for Hospital 1 contained a surrogate measure of congestion, the number of patients at midnight who had earlier been accepted for admission, but who had still not been allocated an inpatient bed (delayed placement patients). The relationship between the number of delayed placement patients and modularity was examined by computing the correlation between their number, and monthly modularity values in the Hospital 1 snapshots. The correlations were examined for networks with, and without, the high coherence modules.

## Results

### The identification of functional structures and network graph characteristics

**Fig. 1:**
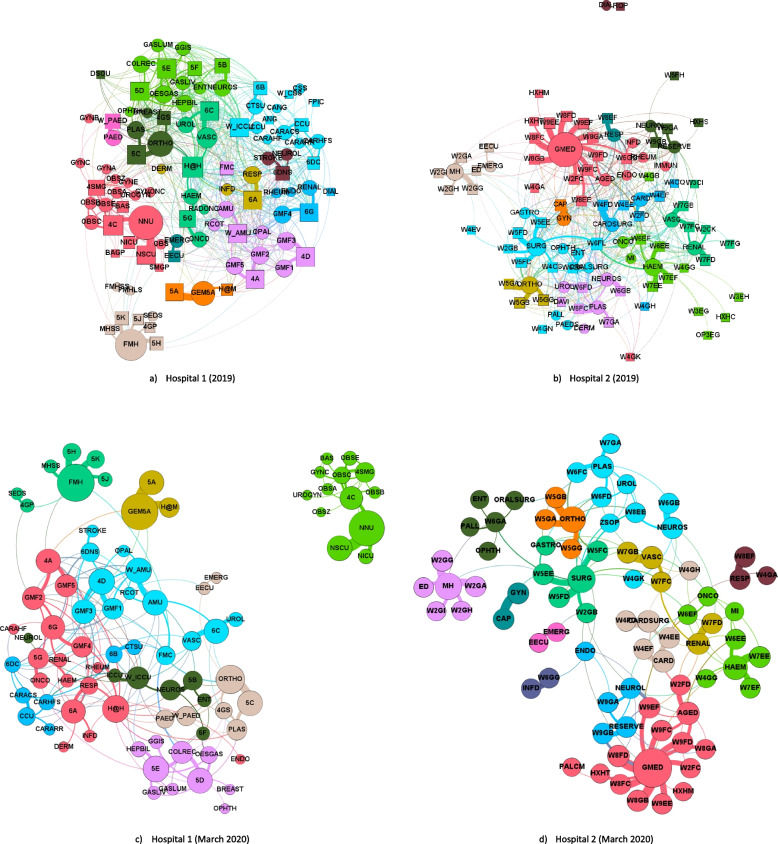
Sample graphs showing modular structures for hospitals 1 and 2 with modules distinguished by colours. Acronyms as used on each node correspond to their original names in the Hospitals. Graphs (**a**) and (**b**) are produced using composite data over the whole of 2019. Nodes corresponding to wards are indicated by squares while clinical units by ovals. Graphs (**c**) and (**d**) are from a single monthly snapshot in March 2020, where both wards and clinical units are indicated by circles. The complete series of graphs, plus the membership of the identified communities, are available on request from the authors

Figure 1 shows two pairs of graphs for each hospital. One of the pairs is the composite graph for 2019, the other is a graph of a monthly snapshot for March 2020. The graphs as shown are taken from a series of graphs that include both individual monthly and composite snapshots for 2019 and 2020. All the graphs are bipartite in form. The nodes are labelled with abbreviations representing either wards or clinical units. The labels identify the usual clinical functions of the modular elements. The size of the nodes represents variations in the numbers of adjacent edges: that is, the degree of the nodes, not their edge weights. Network graphs are topologies. The presentation and format of the graphs can be varied without changing the underlying interactions. The nodes can be rectangles and ovals, or all circles. The colours are applied by the computer program, and the colouring is unique to each generated graph.

**Fig. 2:**
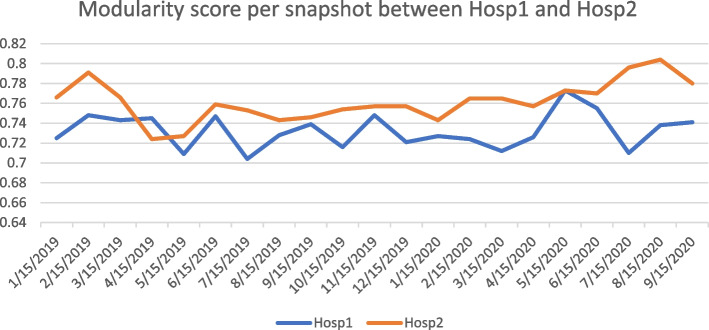
Comparison of modularity scores between two hospitals (the upper one is Hospital 2 while the lower is Hospital 1)

The network graphs confirmed the modular nature of the ward-unit structures through which inpatient care was delivered in both hospitals. The modularity value for the composite graph of Hospital 1 in Fig. 1 (a, 2019) was 0.714 and for Hospital 2 (b, 2019), 0.734. The modularity value of the March 2020 (c, 2020) graph for Hospital 1 was 0.71, and for Hospital 2 (d, 2020) was 0.77. Modular structures were identifiable throughout the whole period of study. Figure 2 shows the monthly modularity scores of the two hospitals up until September 2020.

The 2020 snapshots cover the initial period of the COVID 19 pandemic. Whilst Hospital 1 was involved as a community hospital in the response to the pandemic, Hospital 2 was a designated SARS-CoV-2 response hospital where elective admissions were restricted in anticipation of pandemic demands. At that stage, whilst the morbidity of the SARS-Cov19, a respiratory virus, was not yet clear, it was deemed important to ensure adequate resources were available, both in terms of intensive treatment facilities including ventilators in negative pressure environments, and the staff to support them.

The network graphs appeared to be responsive to the changes in function. In Fig. 2, Hospital 1 showed a brief increase in modularity over the initial COVID-19 pandemic period, whilst Hospital 2 showed a more marked, and more sustained, increase in the same period. ‘Subtraction’ graphs were constructed for both hospitals, in that the adjacency matrices were repopulated entirely with patients admitted only with an emergency designation, or only with an elective designation. It appeared that for Hospital 2 (Fig. 1, d 2020), there was a marked, and fairly brief, overall reduction in admissions, and the residual elective work that was not cancelled was concentrated in a limited number of modules.

**Fig. 3:**
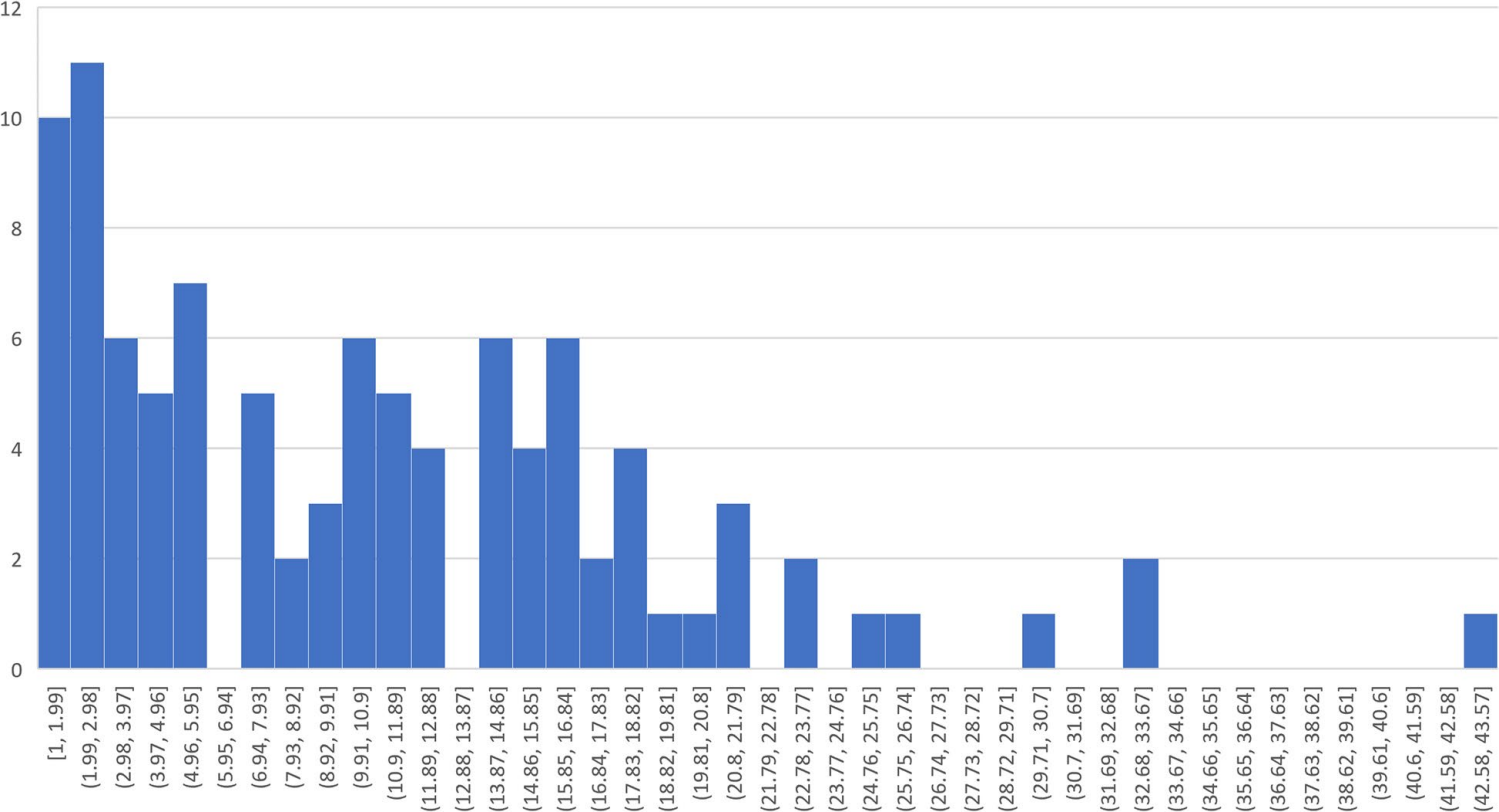
Histogram degree distribution, Hospital 1, 2019 (bin width = 0.99)

Network graphs characterised by the presence of modular interactions between groups of nodes share a number of mathematical properties that reflect important functional characteristics of the systems represented. The degree of a node is the number of other nodes that connect directly to the node in question. As previously described, in graphs where inter-connections between nodes are random, the overall distribution of degrees will be normal, or Gaussian in nature. There will be a mean, or average number of connections, and a low likelihood of extreme numbers of connections. Network graphs with modular structures are complicated systems, and as such, they commonly have a non-linear node degree distribution that is unlikely to have occurred by chance [35]. There is some controversy in the Network Science community over the extent to which complicated graphs share a characteristic node degree distribution in which there are a large number of nodes with a small degree, and a small number of nodes with a much larger degree, the latter nodes forming a fat-tail in a probability distribution [36, 37]. Such distributions may follow a power-law distribution, or some other non-linear fat-tailed distribution [35, 36]. Extensive discussions of the implications of power-law and other distributions are available in [7].

Figure 3 shows the histogram of node degree distribution from Hospital (1) The raw and straight line log-log distributions are clearly non-Gaussian. Analytical calculations using MATLAB [38] confirm that the exponential function is a preferable fit for the distribution. Specifically, the MSE (mean square error) for an exponential type of distribution (Weibull type) was 0.00006, while the error for fitting a normal distribution was 0.0001. Similar findings were identified for Hospital (2) A Weibull distribution is one of the exponential distributions that produces a fat-tail of probabilities.

**Table 1:** Coefficients of variations (CV) of numbers of nodes in modules associated with marker ward-unit node combinations in network graphs of monthly snapshots of Hospital 1 and Hospital 2 patient data

Module	M1	M2	M3	M4	M5	M6	M7	M8
Hospital 1	0.17	0.13	0.14	0.75	0.41	0.41	0.36	0.68
Hospital 2	0.18	0.15	0.18	0.35	0.0.57	0.59	0.47	

### The relationship between network graph identified structures and delayed admissions of emergency patients

In relation to the study of modular structures following the domain expert review, the sub-threshold modules in Hospital 1 included a mental health related module, a women’s health services module and a geriatric assessment unit. In Hospital 2 there was also a mental health unit, an acute medical assessment unit, and a haematology oncology cancer service. In both hospitals, the super-threshold cv modules were the more general acute medical and surgical services.

The relationship between the number of delayed placement patients and modularity was examined by computing the correlation between their number, and modularity in the Hospital 1 snapshots. The number of delayed placement patients varied from a low of 9 to a high of 24. Detailed specification of the numbers of delayed patients per snapshot has not been provided so as to avoid any possibility of a breach of privacy. The month-by-month variations in modularity for Hospital I are shown in Fig. 1. The correlation between the delayed placement numbers and the overall snapshot modularity parameters was not statistically significant (*r* = 0.33).

We re-examined modularity, excluding the three low cv modules, based on the assumption that these services were not intensively involved in the high volume medical and surgical cases that made up the majority of the delayed placement patients [39]. The average modularity score for the remaining snapshots fell from 0.73 to 0.66, with a distribution that ranged from 0.613 to 0.706. The correlation increased to *r* = 0.48 (*p* < 0.05) with lower modularity being linked to increases in numbers of delayed placement patients. We repeated that analysis excluding patients admitted as elective patients in the data set, and the correlation increased slightly to *r* = 0.53 (*p* < 0.05).

## Discussion

A modern general hospital hosts, on a daily basis, a large number of encounters between patients and care providers. Whilst the majority of those encounters have a satisfactory outcome, substantial numbers are adversely affected by a variety of issues [40]. We argued in the introduction that the analysis of healthcare dysfunction has to be linked to an understanding of function, and that a quantitative measure of function may be a structured measure.

In this set of studies, we represented important aspects of the functioning of two general hospitals’ inpatient services as bipartite network graphs. When named wards (and by extension the ward based clinical staff within them) and specified clinical units form the network nodes, and the patients connecting them, the edges, the bipartite graphs represent the clinical structures through which clinical care is delivered in inpatient facilities. The graphs presented here represented the inpatient services of two large Australian public hospitals whose everyday work includes managing large numbers of emergency patients. The graphs covered both a period of usual demand, and the first wave of responses to SARS-CoV-2.

By comparing the modular structures that emerged against random graphs of the interactions between similar numbers of elements we were able to reject the null hypothesis that the processes are represented by random graphs. The identified structures were not the consequence of chance interactions. However, the network modules were the product of a purely ‘arithmetic’ combinatoric procedure. To what extent did the modular systems as represented make sense as representations of the real world systems from which they were derived? That is, whilst the combinatorics might create reliable mathematical structures, were they valid real world representations?

The graphs had face and content validity. All wards, clinical units, and patients were represented. There was a clear pattern to the links between wards and clinical units that was readily interpreted as representing the patterns of care within the hospitals concerned. The patterns did not represent functional structures as multiple isolated ward-unit ‘silos’ [41, 42] or other rigid structures that would shatter when put under strain. Rather, the wards and units fell into a number of distinct but interlinked communities, or modules. The constituent members of the modules were closely interlinked in a variety of ways, but were also linked to other communities or modules, although to a lesser extent, a pattern that conforms to a more general description of a modular system [9]. The modules were heterogeneous, containing a small number of high coherence modules that represented highly specialized services with distinct accession profiles, and a larger number of more general services.

Graphs were generated for each monthly snapshot, and for the accumulated data. The monthly graphs were sparser than the cumulative graphs, which is appropriate for the smaller number of edges in the monthly snapshot graphs. But the underlying modular structures were similar between the monthly and cumulative graphs, and made sense to domain experts. The modularity parameter, a graded, if not parametric, objective measure of the difference between a modular, and matching random graph [18], confirmed the modular nature of the functional structures involved. The modular parameter varied to a limited degree from month to month (Fig. 2), but showed a change in value during periods of changed hospital functioning during COVID based restrictions. Network graphs of healthcare systems that are responsive to changes in the underlying systems of care have a measure of construct validity. The degree distribution of nodes in network graph is an active topic in Network Science [43], and warrants further study in the healthcare context.

Network graphs are state measures. They represent networks at particular points in time. They are descriptive rather than predictive. Nevertheless, we found a relationship between the modularity metric of a graph subsystem focused on the management of medical and surgical emergencies, and the number of emergency patients waiting at midnight for an inpatient bed within the body of the hospital. Substantial number of patients so identified is generally regarded as an index of hospital dysfunction. The Modularity metric represents the movement between organization and randomness, and in Hospital 1, the more random the modular subsystem, the greater the number of patients whose admission was delayed. A causal relationship has not been established, but the possibility that increasing randomness in the organization of relevant functional structures may be predictive of congestion and dysfunction in the relevant system is of considerable interest and requires further study. It may be that system modularity is a candidate metric for a quantitative measure of system functional status.

The graphs confirmed that whilst the modules in the network graphs each had a distinct functional identity, there were a limited number of ‘shortcut’ direct links to other modules within the system. This system characteristic is commonly referred to in Network Science as ‘small-worldness’ [44, 45]. General hospitals with substantial emergency loads need to be able to respond to patients with less common combinations of primary and secondary diagnoses. The ‘small-world’ flexibility of communication in a modular system helps to maintain information and expertise exchange between clinical services without disrupting frequently used functional and clinical pathways.

There are many limitations to these studies. Only two hospitals were involved, and both are part of the same health economy. Only a restricted amount of the very large volumes of data generated by hospitals was used in the studies. Despite having good access to data from diagnostic services, we did not include them in the studies reported here, mainly because their pervasive use means that they shed little light on the kind of structural concerns studied here. Further investigation is warranted. Also, we did not include the totality of emergency services in our analyses. We only included those Emergency Department patients in Hospital who had been designated as inpatients waiting for placement in the body of the hospital. More work is required before this and other healthcare studies can be merged into a whole of hospital connectome [46] of functional interactions.

We restricted our analyses to one combinatoric algorithm. Other forms of analyses are possible, but the Louvain algorithm is widely used [47], and restricting our analysis to one algorithm greatly simplified comparisons between different graphs. We did not extend our studies to the organization of non-clinical [39] services, though we suspect the latter are also likely to be modular in structure.

Whilst managerial oversight in day-to-day processes was not itself readily identifiable in the data we had access to, experience indicates it has an important role in modular structures. When inter-modular incursions appear to be likely to be more extensive than are readily accommodated by existing shortcut paths, senior management intervention may be required to manage friction at the borders [6]. This is not an unfamiliar problem in institutions that run close to capacity, but hard-to- manage inter-modular tensions can be accentuated by external pressures of various kinds. Nevertheless, it may also be the case that the underlying robustness of modular systems allows institutions to rebalance after periods of strain.

### Implications

There is an emerging interest in the application of Network Science to a range of healthcare related issues [48–52]. The current work has a number of implications for health services research and practice. It applies Network Science to a detailed analysis of systems of care within institutions, supplementing existing Network Science studies on movements across healthcare institutions [24, 28].

Network graphs are topologies. They represent interactions irrespective of where they take place. This makes Network Science highly relevant to evaluating and improving the integration of virtual healthcare services with existing (and continuing) intra-mural services, an issue highlighted by responses to the COVID-19 pandemic [53]. Furthermore, with quantitative measures of functional systems in place, attempts to improve access to existing hospital and healthcare services in the face of spikes of externally generated demand can become more nuanced. We have previously described a simulation based strategy for evaluating the impact of variations in demand and access to hospital resources, on hospital congestion episodes [39]. That strategy made a purely empirical distinction between those hospital resources that should be included or excluded from the analysis. Network based studies can put such decisions on a more generalizable footing, enabling institutions to identify the component resources relevant to specific outcomes and plan targeted interventions in areas such as such congestion-related delays. The use of administrative data in both studies means that replications in other settings will be straightforward. Many other opportunities for health service research programs of work will no doubt emerge as researchers and clinicians become familiar with Network Science techniques.

## Conclusion

Bipartite Network Graphs of the organizational and logistic layer of inpatient care systems in two general hospitals were developed using existing healthcare data sources. The graphs consistently represented the hospital structures as modular. The topological characteristics of the network graphs were consistent with those of a range of other complicated technical and social systems [54, 55], implying that the organizational characteristics of hospitals may bear a resemblance to those of other complex systems, an issue that nevertheless requires further research.

Network Graphs are a purely arithmetic product. Their utility rests on their capacity to be meaningfully interpreted in relation to the system from which they are derived. The healthcare graphs were reviewed by independent domain experts with a detailed knowledge of healthcare systems. For each hospital, they found the graphs to be readily interpretable and that they represented systems that contained recognizable specialized modular components, together with a more loosely organized set of general medical and surgical modules. We had hypothesized that the pressure on hospitals’ systems, exerted by variations in demands for care, and by planned reorganizations, would be identifiable within the organizational and logistical middle layer. We were able to confirm this in two ways. First, by demonstrating a relationship between modularity values of subgraphs of the more general medical and surgical services and a hospital-wide congestion indicator. Then, by demonstrating that graph structures were responsive to the changes that occurred during the initial period of the COVID-19 pandemic. The hypothesis testing capacity of the Network graphs in a healthcare context is substantially enhanced by the fact that the values of the modularity metric are derived from tests against random or null state graphs.

Finally, one of the challenges of a Network Graph approach is that the graphs are measures of a hospital state at a point in time. Finding ways for Network Graphs to represent the way networks change [56] is a rapidly developing area of Network Science theory and practice. In this study, we developed a straightforward link between network graphs in a temporal sequence as a prelude to further research on the dynamic aspects of healthcare network graphs.

## Data Availability

The datasets generated and analysed for the current studies were derived from hospital data collections mandated by health care authorities. They contain a variety of routinely collected information, including individual diagnostic and demographic information. For privacy reasons, the primary data sets are not publicly available. Anonymised extracts from the administrative data sets that would allow Network graphs described to be computed are available from the authors on reasonable request.
